# *Pseudobombax parvifolium* Hydroalcoholic Bark Extract: Chemical Characterisation and Cytotoxic, Mutagenic, and Preclinical Aspects Associated with a Protective Effect on Oxidative Stress

**DOI:** 10.3390/metabo13060748

**Published:** 2023-06-13

**Authors:** Tiago Felipe de Senes-Lopes, Jefferson Romáryo Duarte da Luz, Zaira da Rosa Guterres, Eder A. Barbosa, Débora Batista, Ony Araújo Galdino, Marcela Abbott Galvão Ururahy, Elizabeth Cristina Gomes dos Santos, Jorge A. López, Gabriel Araujo-Silva, Maria das Graças Almeida

**Affiliations:** 1 Postgraduate Program in Health Sciences, Center for Health Sciences, Federal University of Rio Grande do Norte, Natal 59012-570, RN, Brazil; seneslopestf@gmail.com (T.F.d.S.-L.); onygaldino@gmail.com (O.A.G.); 2 Multidisciplinary Research Laboratory, Department of Clinical and Toxicological Analysis, Health Sciences Center, Federal University of Rio Grande do Norte, Natal 59012-570, RN, Brazil; jefferson.luz@ueap.edu.br (J.R.D.d.L.); deborabioquimica@gmail.com (D.B.); elizabethcgsantos@gmail.com (E.C.G.d.S.); jorgejal@gmail.com (J.A.L.); 3 Laboratory of Cytogenetics and Mutagenesis, State University of Mato Grosso do Sul, Mundo Novo 79980-000, MS, Brazil; zairaguterres@yahoo.com.br; 4 Organic Chemistry and Biochemistry Laboratory, State University of Amapá, Macapá 68900-070, AP, Brazil; gabriel.silva@ueap.edu.br; 5 Laboratory of Synthesis and Analysis of Biomolecules, Institute of Chemistry, University Campus Darcy Ribeiro, University of Brasília, Brasília 70910-900, DF, Brazil; bioederr@gmail.com; 6 Postgraduate Program in Pharmaceutical Sciences, Center for Health Sciences, Federal University of Rio Grande do Norte, Natal 59012-570, RN, Brazil; marcela.ururahy@ufrn.br

**Keywords:** caatinga biome, *Pseudobombax parvifolium*, oxidative stress, traditional medicine

## Abstract

Plants have long been used in traditional medicine to treat illnesses. Nevertheless, their chemical diversity requires studies to establish the extract dosage and its safe use. *Pseudobombax parvifolium*, an endemic species of the Brazilian Caatinga biome, is commonly used in folk medicine, due to its anti-inflammatory properties related to cellular oxidative stress; however, its biological properties have scarcely been studied. In this study, we chemically characterized the *P. parvifolium* hydroalcoholic bark extract (EBHE) and evaluated its cytotoxic, mutagenic, and preclinical aspects, as well as its antioxidant effect. Our phytochemical analysis revealed a significative total polyphenol content and identified loliolide for the first time in this species. Cytotoxicity, mutagenicity, and acute oral and repeated dose indicated no toxic effects on cell culture, *Drosophila melanogaster*, and Wistar rat exposure to different EBHE concentrations, respectively. Furthermore, we observed a significant decrease in lipid peroxidation and a mild hypoglycemic and hypolipidemic effect with repeated oral dosing of EBHE. Although there were no significant changes in glutathione content, we did observe a significant increase in superoxide dismutase at a dose of 400 mg/kg and in glutathione peroxidase at doses of 100, 200, and 400 mg/kg. These findings suggest that EBHE has potential as a source of bioactive molecules, and it can be used safely in traditional medicine and in the development of herbal medicines for application in the public health system.

## 1. Introduction

Medicinal plants are used worldwide by humans as therapeutic resources and as an alternative to conventional medicine in contemporary society. Currently, these natural products play a pivotal role as a source of compounds, and many drugs derived from traditional herbal medicine are used in modern pharmacotherapy [1]. Despite the increasing commercialization of herbal remedy as safe for consumers, these plant materials require monitoring due to their potential adverse and toxic effects. Given this concern, medicinal plants have become the focus of scientific studies due their chemical diversity, which can have unknown biological effects. Therefore, quality control is crucial in order to evaluate the safety, efficacy, and validation of medicinal plant use, with regard to the potential risks associated with their chemical composition [2].

In this context, Brazil boasts a comparatively rich biodiversity, corresponding to approximately 20% of the biodiversity in the world. This ecosystem has over 55,000 cataloged native plant species, which, associated with ethnopharmacological knowledge, provides valuable information about their diverse application [3]. Concerning the Caatinga biome in the Brazilian northeast, its biodiversity is promising since it harbors a significant flora with about 20,000 species. Although scarcely explored, this biome deserves attention due to its clinical–pharmaceutical potential and the ecosystem protection against degradation caused by the agricultural and intense extractive activity [4].

In Brazil, the Malvaceae family is represented by about 13 genera and 80 species, whose distribution occurs mainly in the north and northeast regions [5]. Accordingly, *Pseudobombax parvifolium* was selected for this study since it is one of the most representative of the Malvaceae family and is an endemic species of the Caatinga biome in northeastern Brazil [6].

In traditional medicine in northeastern Brazil, *P. parvifolium* is widely used for the treatment of urinary tract infections and spine inflammation, and to relieve symptoms resulting from ulcers and gastritis [7]. Studies have reported the use of plants from the Malvaceae family for substance discovery and therapeutic purposes [8]. Data on the pharmacological properties of the ethanolic bark extract of *Pseudobombax marginatum* have shown anti-inflammatory, antipyretic, and analgesic effects, which are correlated to the flavonoid content detected in the extract [9]. Regarding the antimicrobial activity of *P. marginatum* bark, few reports have described this effect. Santos et al. [10] showed the bacteriostatic effect of 70% ethanolic bark extract on *Candida albicans* and on Gram-positive and -negative bacteria. Furthermore, chemical analysis of this extract identified catechin as the major compound, which is associated with antimicrobial activity.

Despite the widespread use of Malvaceae species from the Caatinga biome in folk medicine, there is poor scientific evidence reporting their chemical composition and potential impacts on human health [11,12]. Overall, these plants may be the only form of treatment available for populations in many locations. Therefore, obtaining information regarding their constituents and validating their uses are pivotal to provide data on their safe consumption, as well as avoiding indiscriminate use. The lack of phytochemical knowledge, therapeutic value, risk, and toxicity can cause intoxication and even death, becoming a serious public health problem [13]. Concerning *P. parvifolium*, this study is important since it provides phytochemical and biological information about the medicinal potential of this species to subsidize pharmacological research based on traditional uses.

Plants produce a wide variety of molecules and highly specific secondary metabolites (e.g., phenolic compounds, and terpenes), which play several roles in plant growth and development processes. Furthermore, these compounds display positive beneficial effects on human health and agricultural production, arousing scientific interest due to their potential application in several industrial areas, and significantly contributing to the economy [14,15]. Among them, the pharmaceutical industry stands out, as plant secondary metabolites are a promising source of new molecules for drug research and development, with potential for clinical use [16].

Nevertheless, many of these chemical compounds can interact with DNA and other biomolecules, promoting alterations at the biochemical, physiological, and molecular levels [17]. Despite its common use in traditional medicine, no report has described the evaluation of adverse effects of the *P. parvifolium* hydroalcoholic bark extract. In this context, results of the present study can provide data regarding the phytocomposition and potential toxic, cytotoxic, and mutagenic effects, highlighting this plant as a source of biomolecules with clinical–therapeutic application [15]. Accordingly, this study analyzed the phytochemical composition of the *P. parvifolium* hydroalcoholic bark extract, and evaluated the cytotoxic, mutagenic, and acute and repeated dose oral toxicity effects of this extract, using in vitro and in vivo experimental models.

## 2. Materials and Methods

### 2.1. Materials and Reagents

Aluminum chloride, sodium acetate, chloral hydrate, potassium phosphate, sodium chloride, 1,1,3,3-tetraethoxypropane, Folin–Ciocâlteu reagent, Dulbecco′s modified Eagle′s medium (DMEM), fetal bovine serum (FBS), gentamicin sulfate, penicillin G sodium, glycerol, gum arabic, ketamine-D4 hydrochloride solution, xylazine hydrochloride, gallic acid, and quercetin were obtained from Sigma-Aldrich (São Paulo, Brazil). Ethanol was obtained from Merck (São Paulo, Brazil). AlamarBlue^®^ reagent and MTT (3-(4,5-dimethylthiazol-2-yl)-2,5-diphenyltetrazolium bromide) were purchased from Invitrogen (Carlsbad, CA, USA). Doxorubicin hydrochloride (DXR) was obtained from Biossintética (São Paulo, Brazil). Bacteriological agar and nipagin were obtained from ACS Científica (São Paulo, Brazil). All chemicals and reagents were analytical grade.

### 2.2. Collect and Plant Identification

*P. parvifolium* barks were collected from natural populations in Vale do Açu, Municipality of Alto do Rodrigues, State of Rio Grande do Norte/Brazil (5°15′22.7″ S 36°43′00.3″ W) by SisGen (Registration No.A6770C7) and SisBio (License No.400606) authorizations. The taxonomic identification was performed by Dr. Jefferson G. de Carvalho-Sobrinho, depositing a voucher specimen (No. UFRN8204) in the Department of Botany and Zoology Herbarium of the Federal University of Rio Grande do Norte.

### 2.3. Preparation of the P. parvifolium Hydroalcoholic Bark Extract

After collection, the plant material was separated and air-dried at 40 °C for 72 h. Then, it was pulverized to a granulometry <180 and stored in amber containers before extract preparation. The powdered bark was extracted by maceration according to the methodology adapted from the Brazilian Pharmacopoeia [18] under the following conditions: 1 g of vegetable sample/5 mL of solvent (1:5, *p*/*v*) in an ethanol/water (50:50, *v*/*v*) for 7 days at room temperature with periodic stirring. The extract was filtered, concentrated by rotary evaporation at 40 °C, and subsequently lyophilized. This extract was denominated as EBHE.

### 2.4. Determination of Total Phenolic Content (TPC)

TPC was determined in triplicate according to the modified Folin–Ciocâlteau method described by Georgé et al. [19]. Briefly, a 25 μL aliquot of EBHE (2000 μg/mL) diluted in ethanol was mixed with 125 μL of Folin–Ciocâlteu reagent diluted in water (10×) and 150 μL of distilled water. After 5 min, 100 μL of 7.5% Na_2_CO_3_ solution was added, and the reaction system was mixed vigorously and incubated (15 min/50 ± 2 °C). Then, the absorbance was measured against the blank at 760 nm in a microplate ELISA reader (Epoch-BioTek, Winooski, VT, USA). The TPC was calculated by a gallic acid calibration curve (2.5, 5, 10, 25, 50, 75, and 100 μg/mL; R^2^ = 0.9978), and the results were expressed as mean ± SEM of milligrams of gallic acid equivalents per mg of dry EBHE (mg GAE/g dry EBHE).

### 2.5. Determination of Total Flavonoid Content (TFC)

TFC was determined in triplicate using the aluminum chloride colorimetric method described by Silva et al. [20]. Briefly, 50 µL aliquot of EBHE (2000 µg/mL) diluted in ethanol was mixed with 160 µL of ethanol, 20 µL of AlCl_3_ solution (1.8%, *w*/*v*), and 20 µL of sodium acetate (8.2%, *w*/*v*). After incubation (40 min/25 ± 2 °C/darkness), the absorbance was measured against a blank at 415 in a microplate ELISA reader (Epoch-BioTek, Winooski, VT, USA). The TFC was calculated from the quercetin calibration curve (1.25, 5, 10, 25, 50, 75, 100, 150.0, and 200 μg/mL; R^2^ = 0.9921), and the results were expressed as the mean ± SEM of milligrams of quercetin equivalents per mg of dry EBHE (mg QE/g dry EBHE).

### 2.6. Ultra Liquid Chromatography Coupled to Mass Spectrometry (LC–MS/MS)

LC–MS/MS was used for EBHE chemical characterization. Extract samples were reconstituted and homogenized in methanol, centrifuged at 13,000 rpm for 30 min, and filtered through a 0.22 mm membrane; the supernatants were stored at −20 °C. After diluting a supernatant aliquot in the mobile phase (pure acetonitrile), the EBHE was analyzed by LC–MS/MS according to the methodology described by Luz et al. [21]. The analysis was performed on an UHPLC ultra-LC 110-XL Eksigent (AB Sciex, Framingham, MA, USA) equipped with a Kinetex 2.6 µm HILIC 100 Å, LC column (30 × 2.1 mm) coupled to a Nexera-Mass Spectrometer TripleTOF 5600+ (AB Sciex, Framingham, MA, USA). Before starting the analysis, the column was equilibrated with a 5% acetonitrile/0.1% formic acid water solution, maintaining the column temperature at 40 °C. Then, 2 µL of the sample was automatically injected. The separation was performed by applying a linear gradient of 5% acetonitrile/0.1% formic acid ranging from 5% to 95% for 10 min at a flow rate of 0.4 mL/min at 40 °C. The mass spectrometer operated in positive ionization mode (IDA) to monitor ions in the mass range of 100 to 1800 *m*/*z* and source temperature of 650 °C. The IDA mode acquisition was configured to fragment ions of *m*/*z* 100–1250, whose charges ranged from 1 to 3, exhibiting an intensity greater than 1000 counts. The other parameters were configured under the following conditions: pulse frequency = 15.392 kHz, curtain gas = 15.000 psi, ion source gas 1 = 50.000 psi, ion source gas 2 = 45.000 psi, accumulation time = 250.0 ms, period cycle time = 900 ms, and floating ion spray voltage = 5500 V. Additionally, data referring to a blank control were acquired. Initially and in every five LC–MS/MS analyses, the spectrometer was calibrated using standard calibration solution to validate the accuracy of approximately 0.5 ppm (sodium iodide (2 µg/µL) and cesium iodide (50 ng/µL) in 50/50 2-propanol/water).

LC–MS/MS mass data were converted to mzXML and submitted for analysis by the Molecular-Library Search-V2 tool (release_14 version) and then analyzed using the GNPS (Global Natural Product Social Molecular Networking) online database (http://gnps.ucsd.edu, (accessed on 25 April 2023). Data were filtered to remove peaks with ~17 Da, related to Molecules 2022, 27, and 1084, and 13 of 17 of the *m*/*z* values of precursors in the MS/MS spectra for selecting just the top six peaks in a 50 Da window across the spectrum. Subsequently, data were clustered by MS-Cluster with tolerances for an original mass of 0.02 Da and an MS/MS fragment ion of 0.1 Da in order to create consensus spectra. Consensus spectra with fewer than two spectra were eliminated for analysis in the GNPS spectral libraries. Library spectra were filtered according to the input data. During analysis, correspondences were maintained between network and library spectra that exhibited a score greater than 0.85 and at least four matching peaks. Substances were considered identified in the sample if the mass spectra obtained at least four ions that match; a cosine score > 1 represented identical spectra, while a score of 0 indicated no similarity [21].

### 2.7. Cell Culture

African green monkey renal epithelial cells (Vero) [ATCC CCL-81], human embryonic kidney cells (HEK-293) [ATCC CRL-1573], mouse fibroblast cells (3T3) [ATCC CL-173], and rat liver epithelial cells (BRL-3A) [ATCC CRL-1442] were obtained from the American Type Culture Collection (Rockville, MD, USA). Cells were grown in DMEM, supplemented with 10% FBS, 100 IU/mL of sodium penicillin, and 40 μg/mL of gentamicin sulfate in a humidified atmosphere containing 5% CO_2_ at 37 °C. These cell lines were selected on the basis of their susceptibility and wide use to evaluate cytotoxicity induced by chemical components [22].

### 2.8. MTT Cell Viability Assay

The 3T3, HEK293, Vero, and BRL-3A cells were individually challenged in triplicate to increasing concentration of EBHE (0.1, 1, 10, 100, and 1000 µL/mL) to assess cell viability. Cells were seeded in 96-well microplates (1 × 10^4^ cells/well) in DMEM supplemented with 10% FBS and incubated at 37 °C in 5% CO_2_ to reach confluency. After 24 h, the medium was removed, before adding 100 µL of MTT reagent (1 mg/mL) to each well, and the system was incubated for 4 h. Then, the supernatant was discarded, and 100 µL of DMSO was added to dissolve formazan crystals before monitoring cell viability at 570 nm using a microplate ELISA reader [23] (Epoch-Biotek, Winooski, VT, USA). Cells grown in DMEM alone were used as a negative control.

### 2.9. Alamar Blue^®^ Viability Assay

The EBHE cytotoxic effect on 3T3, HEK293, VERO, and BRL-3A cells was evaluated in triplicate by the Alamar Blue^®^ assay. Briefly, cells were seeded in 96-well microplates (1 × 10^4^ cells/well) in DMEM supplemented with 10% FBS and incubated at 37 °C in 5% CO_2_ for 24 h to promote adhesion. After cell exposure to increasing EBHE concentrations (0.1, 1, 10, 100, and 1000 µL/mL) for 24 h at 37 °C, 10% of Alamar Blue^®^ reagent was added to each well, corresponding to a volume equivalent to a volume of culture medium. Cells were incubated again (4 h/37 °C/5% CO_2_) before removing culture medium to add 100 μL of DMSO and assessing cell viability at 600 nm in a microplate ELISA reader [24] (Epoch-Biotek, Winooski, VT, USA). Cells grown in DMEM alone were used as a negative control.

### 2.10. Mutagenicity Assessment

*Drosophila melanogaster* strains (fruit fly) stocks, as well as the crosses carried out, were kept in sterile flasks containing an alternative culture medium for *Drosophila* (820 mL of water, 11 g of agar, 156 g of banana, 1 g of nipagin and 25 g of substrate for yeast). Cultures were kept at 25 ± 2 °C and 60% ± 5% humidity in a BOD (biological oxygen demand) chamber with a 12 h light/dark photoperiod.

### 2.11. SMART (Somatic Mutation and Recombination Test)

The *D. melanogaster* SMART assay is based on the loss of heterozygosity and detects various genetic events such as mutation, deletion, translocation, and mitotic recombination [25]. Thence, two crossings were performed: (1) standard cross (ST) established by mating virgin flare^3^ females (*flr^3^*/*In(3LR) TM3*, *ri ppsep l (3)89Aa bx34ee Bd^S^*) with multiple wing hairs males (*mwh*/*mwh*); (2) high bioactivation (HB) cross mating ORR/flare^3^ virgin females (*ORR/ORR*; *flr^3^*/*In(3LR) TM3*, *ri ppsep l (3)89Aa bx34ee Bd^S^*) with multiple wing hairs males (*mwh*/*mwh*). From both crosses, offspring were obtained carrying two genotypes: (1) *mwh+*/*+flr^3^* (marker-heterozygous [MH]), one trans-heterozygous for the recessive markers *mwh* and *flr^3^*, with normal round wings; (2) *mwh+*/*TM3*, *Bd^S^* (balancer-heterozygous [BH]), a heterozygote for the *TM3* balancer chromosome, with serrated wings [26].

Larvae for the assay were obtained by keeping females involved in crossings in glass flasks containing a layer of fresh biological yeast for 8 h to induce oviposition. A solid agar base supplemented with yeast and sucrose was prepared 24 h before oviposition. Adult flies were removed from flasks, while third-stage larvae (72 ± 4 h) were collected and then transferred to containers containing a culture medium prepared with 1.5 g of instant mashed potatoes as alternative *Drosophila* medium (Yoki S.A., São Bernardo do Campo, São Paulo, Brazil) hydrated with 3 mL of increasing EBHE concentrations (1.25, 2.5, and 5.0 mg/mL). Distilled water and doxorubicin (DXR-0.125 mg/mL) were used as negative and positive controls. The different EBHE concentrations, as well as the negative and positive controls, applied in this assay were based on a previous study with plant extracts [27]. All larvae kept in the flasks until adulthood were collected, counted, and transferred to tubes containing 70% ethanol. Forty replicates of each treatment were used in independent experiments.

For mutant spot analysis under an optical microscope with a magnification of 400×, pairs of wings were detached from the body and placed side by side on histological slides using Faure’s solution [27]. Genetic alterations were examined according to the number and type of spot: small single spots (one or two mutant trichomes presence witch *mwh* or *flr^3^* phenotypes), large single spots (presence of *mwh* spots or only *flr^3^* with more than three mutant trichomes), and twin spots (presence of *mwh* and *flr^3^* mutant trichomes) [26].

### 2.12. Animals

Wistar rats (250–300 g, 2 months old, both sexes) were supplied by UFRN Health Sciences Center Bioterium for oral toxicity tests. Animals were kept in polypropylene cages (n = 5) during adaptation and experimentation periods under standard environmental conditions (25 ± 2 °C/60% ± 5% humidity/12-h light/dark photoperiod) with food and water *ad libitum*. Experimental procedures were performed according to Brazilian College of Animal Experimentation guidelines, Universal Declaration of Animal Welfare, Ethical Principles in Animal Research and the UFRN Ethics Committee for Use of Animals (certificate No.173.006/2019).

### 2.13. Acute Oral Toxicity

The current oral acute toxicity assay was performed according to the National Health Surveillance Agency method [28] and guideline No. 423 of the Organization for Economic Co-Operation and Development [29]. Male Wistar rats (60 days old) were divided into two groups (n = 3), a group treated with a single EBHE oral dose of 2000 mg/kg, the limit dose recommended by the OECD regulatory guidelines, compared to the control group receiving only distilled water for 14 days. The single dose resuspended in water was administered by gavage, not exceeding 1 mL/100 g of animal body weight. The animal number used in the study followed OECD recommendations that indicate the smallest possible animal number to obtain statistical significance.

After oral administration, animals were observed periodically for 3 h, intermittently for the next 12 h, and then once every 6 h during 14 days for clinical, mortality, behavioral, and neurological changes, or other abnormalities (e.g., vocal frenzy, tremors, piloerection, cramps, diarrhea). Water consumption and feed intake were monitored at 2 day intervals, while body weight was checked every 7 days until the trial end. All signs evaluated after Hippocratic screening were recorded for further investigation.

On the 14th day, the animals were anesthetized intraperitoneally with xylazine:ketamine (1:1), euthanized, and laparotomized for pooled blood collection of each group by cardiac puncture and evisceration. Organs (liver, kidney, spleen, lung, heart, and stomach) were removed for macroscopic and relative weight determinations. Livers were washed with 0.9% NaCl for further oxidative stress parameter evaluation according to Batista et al. [30].

### 2.14. Repeated Dose Toxicity

The repeated dose oral toxicity procedure was performed according to the methodology proposed in the OECD guideline No. 407 [31], while the concentration of extract doses was established according to Batista et al. [30]. Male and nulliparous female Wistar rats (60 days old) were divided into four groups (n = 5) corresponding of three treated groups with different doses of EBHE (100, 200, and 400 mg/kg) and control group received only distilled water over 28 consecutive days.

After oral administration, rats were monitored continuously for 3 h, periodically for the next 12 h, and then once every 6 h for the next 28 days for clinical observations, mortality, behavioral, neurological, or other abnormalities (e.g., frenzy, vocal tremors, piloerection, cramps, and diarrhea). Water consumption and feed intake were checked at 2 day intervals, and weight was every 7 days until the trial end. Animal signs evaluated after Hippocratic screening were registered for further investigation.

At the 28th day, animals were anesthetized intraperitoneally with xylazine:ketamine (1:1) and then euthanized and laparotomized for pooled blood collection of each group by cardiac puncture for biochemical analysis, and evisceration. Organs (liver, kidney, spleen, lung, heart, and stomach) were removed for macroscopic and relative weight determinations. Livers were washed with 0.9% NaCl for further oxidative stress parameter evaluation according to Batista et al. [30].

### 2.15. Biochemical Parameters

Serum pool samples from each experimental group of animals were used for further biochemical analyses to quantify uric acid, direct bilirubin (DB), indirect bilirubin (IB), total bilirubin (TB), cholesterol (Chol), glucose (Gluc), triglycerides (Trig), urea, aspartate aminotransferase (AST), alanine aminotransferase (ALT), and gamma-glutamyl transferase (γ-GT). The determinations used commercial Labtest kits (Labtest^®^, Labtest Diagnóstica S.A., Vista Alegre, Brazil) according to the manufacturers’ instructions in an automatic LabMax Plenno automated biochemical analyzer (Labtest^®^, Labtest Diagnóstica S.A.).

### 2.16. Preparation of Liver Homogenate

Liver tissue (1 g) from each experimental group was homogenized in cold 20 mM potassium phosphate buffer (pH 7.4) and homogenized in a Potter–Elvehjem homogenizer, obtaining a 10% (*w*/*v*) homogenate, as described by Batista protocol et al. [30] with modifications. Afterward, the homogenate was centrifuged (4000 rpm/4 min/4 °C), and the supernatant was used to evaluate glutathione (GSH), glutathione peroxidase (GPx), superoxide dismutase (SOD), and thiobarbituric acid reactive substances levels (TBARS).

### 2.17. Oxidative Stress Analysis

Lipid peroxidation was determined at 553 nm by TBARS concentration (mmol/L) according to the colorimetric method described by Yagi [32]. Concerning GSH quantification µmol/L, its determination at 420 nm was performed according to the method described by Beutler et al. [33]. The SOD (IU/mg protein) and GPx (IU/mg protein) activities were determined at 510 and 340 nm, respectively, according to Ransel commercial kit (Ransel Laboratories Ltd., Crumlin, UK).

### 2.18. Statistical Analysis

Statistical analysis was performed using GraphPad Prism Software version 5.0 (San Diego, CA, USA). Toxicity, biochemical parameters, and oxidative stress data were initially analyzed for normal distribution using the Kolmogorov–Smirnov test. Results from each experiment were compared to their matched control group using Student’s *t*-test with ANOVA and a post hoc Tukey–Kramer test. The analyzed variables were expressed as the mean ± standard deviation, and a *p*-value of less than 0.05 (*p*  <  0.05) was considered statistically significant.

Concerning mutagenicity using SMART assay, statistical analysis was determined according to the Frei and Wrügler method [34] to differentiate by type and size of mutant spots, and analyzed using the two-tailed chi-square test of Kastenbaum and Bowman [35] for proportions, with a significance level of α = β = 5% (*p* < 0.05), where a statistical diagnosis was positive (+), negative (−), or inconclusive (i).

## 3. Results

The hydroalcoholic extract of *P. parvifolium* bark (EBHE) exhibited a yield of 8.92%. The total phenolic compound was 24.26 mg GA per g of dry extract and total flavonoid content was 16.84 mg QE per g of dry extract. These data provide valuable information about the chemical composition of the extract, specifically the presence of phenolic compounds and flavonoids.

**Figure 1:**
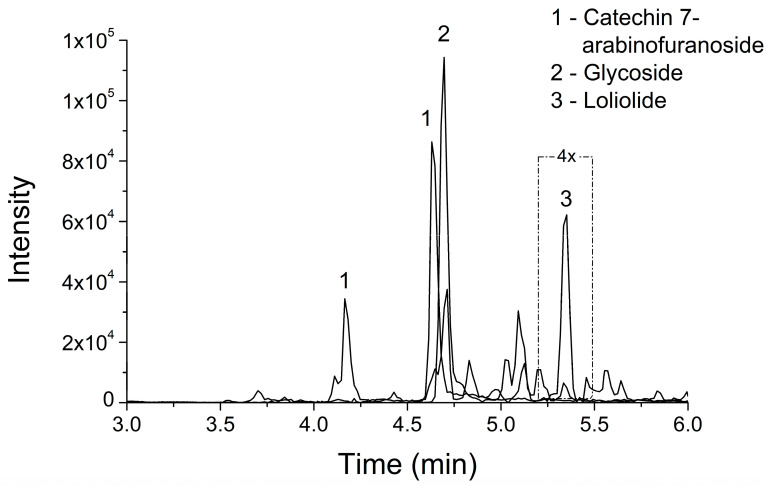
LC–MS/MS fingerprint of *P. parvifolium* hydroalcoholic bark extract (EBHE) showing an extracted ion chromatogram for the ions: (**1**) *m*/*z* 423,13-(2R,3S)-7-[(2S,3R,4R,5S)-3,4-dihydroxy-5-(hydroxymethyl)oxolan-2-yl]oxy-2-(3,4-dihydroxyphenyl)-3,4-dihydro-2H-chromene-3,5-diol; (**2**) *m*/*z* 600,265-2-[[5-(4-hydroxy-3,5-dimethoxyphenyl)-6,7-bis(hydroxymethyl)-1,3-dimethoxy-5,6,7,8-tetrahydronaphthalen-2-yl]oxy]-6-(hydroxymethyl)oxane-3,4,5-triol; (**3**) *m*/*z* 197,116-loliolide. The whole chromatogram (until 10 min) can be visualized in the Supplementary Materials.

LC–MS/MS analysis of EBHE in positive mode was conducted to further characterize its chemical composition, specifically focusing on polyphenols due to prior knowledge of their presence in the extract. The MS/MS spectra of the detected compounds were putatively identified using the GNPS database, considering only spectra with cosine ≥0.85 and mass difference ≤0.005. Figure 1 depicts the EBHE phytochemical profile, indicating three identified and acquired chromatographic peaks, corresponding to possible structures deposited in the GNPS database, identified from the analysis of peaks of fragments generated by mass spectrometry. Table 1 provides information on the putatively identified phytocomponents and their respective cosines, mass differences, and masses. The identification of these compounds in the genus *Pseudobombax* is novel, as there are limited phytochemical studies on species of this genus in the literature.

**Figure 2:**
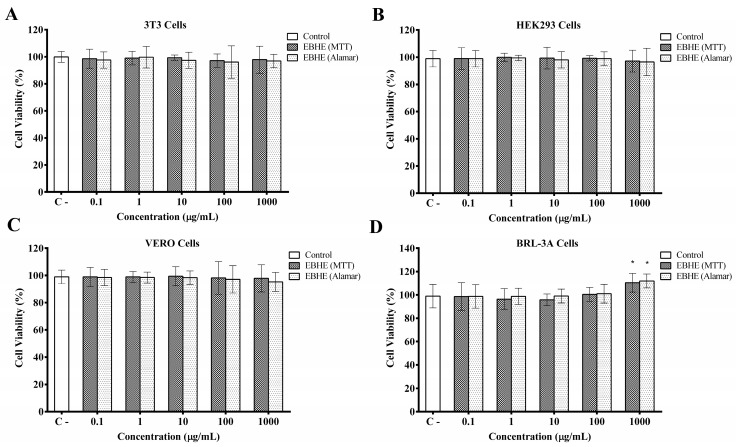
Cytotoxicity effects of *P. parvifolium* hydroalcoholic bark extract (EBHE) on (**A**) mouse fibroblast cells (3T3), (**B**) human embryonic kidney cells (HEK293), (**C**) African green monkey kidney epithelial cells (VERO), and (**D**) rat liver epithelial cells (BRL-3A). Cell viability was measured by the MTT and Alamar Blue assays. A DMEM culture was used as a negative control for cytotoxicity. * *p* < 0.05; values are significant compared to the control group.

Figure 2 depicts the EBHE effect on cell viability assessed using the in vitro MTT and Alamar Blue^®^ assays. The potential cytotoxicity of this extract was evaluated in cultured mouse fibroblast cells, human embryonic kidney cells, African green monkey renal epithelial cells, and rat liver epithelial cells after challenging these cells for 24 h at different concentrations of EBHE. Regarding the MTT assay, cell viability is detected on the basis of oxidative metabolism; a slight induction, although significant, was only observed in the proliferation of rat liver epithelial cells at the concentration of 1000 µL/mL of EBHE compared to the control (Figure 2D). Likewise, an increase in rat liver epithelial cell proliferation was verified by the Alamar Blue^®^ at the highest extract concentration. No cytotoxic effects were observed at the other concentrations of extracts when all cells were exposed to different concentrations.

Regarding the survival of third-stage larvae exposed to different EBHE concentrations, results revealed a high rate of survival of adults at all evaluated concentrations, with no differences between the number of survivors of negative control and the number of survivors in all tested concentrations (1.25, 2.5, and 5 mg/mL) of EBHE. Therefore, this extract displayed no toxic effect on *D. melanogaster* larvae exposed to the experimental conditions evaluated compared to the negative control.

Table 2 shows the results referring to the evaluation of the EBHE mutagenic potential through the mutagenic activity and somatic recombination test in *Drosophila melanogaster*, applying the SMART assay. Results indicated no mutation induction caused by different extract-tested concentrations. Doxorubicin hydrochloride used as the mutagen inducer in the positive control revealed positive results for a single small spot, single large spot, twin spot, and total spots. On the other hand, the negative control exhibited the lowest results at all frequencies of stains, enabling the experiment. The three EBHE concentrations used experimentally displayed negative results in the total of stains. In the HB crossing analysis, it was observed that the frequency and number of spots were higher than those observed in the ST crossing. Overall, these data indicated no mutagenicity after the larva exposure to different concentrations of EBHE.

For the acute and sub-chronic oral repeated daily 28 day dose toxicities in rodents, the animal behavior was observed systematically, providing an overall estimate about the toxicity presented immediately after administration of EBHE, during the subsequent hours, after 24 h, and after 14 days of treatment for the acute toxicity and 28 days of treatment for the repeated extract dose. Animals from both the control group and the EBHE-treated groups showed no detectable clinical changes, whether immediately after compound administration or over the treatment period. No significant differences were observed between the EBHE-treated groups either.

The animal weight was also periodically monitored throughout the experimental procedure. No significant difference was registered between the initial and final periods of treatments. Table 3 displays the results of the relative weights of animal organs. No statistically significant differences in organ weights were observed between the EBHE-treated animals for 14 days (acute) and 28 days (repeated dose) compared to the respective control group.

Furthermore, biochemical parameters were analyzed using the blood of animals from the study of acute oral toxicity (14 days) and repeated dose oral toxicity (28 days), as shown in Table 4. No alteration was observed in blood concentration for most of the biochemical parameters assessed in EBHE-treated groups during 14 and 28 days. Nonetheless, the levels of urea decreased significantly in animals from the EBHE-treated groups subjected to the acute oral and repeated dose oral toxicity tests compared to the respective control groups.

**Figure 3:**
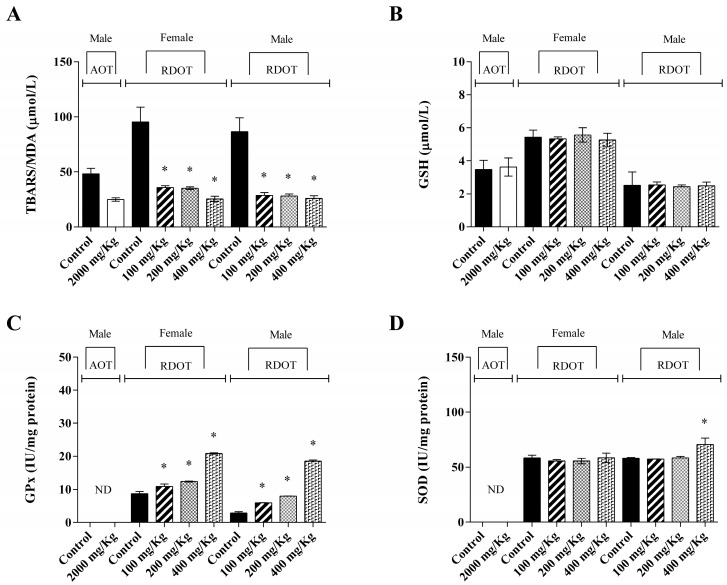
Effects of *P. parvifolium* hydroalcoholic bark extract (EBHE) on antioxidative parameters and lipid peroxidation (TBARS) in experiments of acute and repeated dose oral toxicities in liver tissue of Wistar rats: (**A**) thiobarbituric acid reactive substances levels (TBARS); (**B**) glutathione (GSH) levels; (**C**) glutathione peroxidase (GPx) activity; (**D**) superoxide dismutase (SOD) activity. Values are presented as the mean ± SD. Acute oral toxicity (n = 3); repeated dose oral toxicity (n = 5). Comparisons between groups were analyzed with ANOVA and Tukey’s post hoc test. Malondialdehyde (MDA), reduce glutathione (GSH), superoxide dismutase (SOD), glutathione peroxidase (GPX). * *p* < 0.05; values are significant compared to the control group of the same experiment. AOT: acute oral toxicity; RDOT: repeated dose oral toxicity.

With regard to the antioxidant parameters analysis, Figure 3 exhibits the results of the liver homogenate evaluation from EBHE-treated animals. In repeated dose oral toxicity, GPx activity showed a significant increase in EBHE-treated groups, while, for SOD activity, a significant increase was only observed in male rats treated with 400 mg/kg EBHE, in both cases compared to the group control. Concerning GSH levels, no statistically significant difference was observed in the EBHE-treated groups in acute toxicity or in repeated dose oral toxicity compared to the respective control groups. No activities related to SOD and GPx were determined in animals from the acute toxicity assay. On the other hand, exposure to different doses of EBHE showed a marked decrease in TBARS concentration in treated animal groups with values of approximately 50% and 70% for acute and repeated dose oral toxicities assays, respectively, compared to control groups.

## 4. Discussion

For millennia, medicinal plants have been a worldwide practice intrinsically associated with traditional medicine. Thus, natural therapeutic resources are among nature’s oldest contributions to human wellbeing. This use has become an important alternative for primary healthcare in economically developing countries [36]. Furthermore, in recent decades, a significant worldwide increase in herbal product consumption has been registered for primary healthcare, as well as plant screening to explore their bioactive compound diversity, considering the role of natural products in drug development. Today, many active principles derived from plants are used in several drugs developed by the pharmaceutical industry [37].

Therefore, ethnopharmacology as a scientific approach provides traditional knowledge about the biological activities of plant extracts, allowing a pharmacological–toxicological study of these preparations [38]. Considering the ethnopharmacological context associated with the scarce information about *P. parvifolium*, this study is unprecedented since it focuses on its traditional use as an anti-inflammatory, antinociceptive, and antimicrobial [9,10]. Despite the poor phytochemical data, studies have identified secondary metabolites of therapeutic importance in the Malvaceae family, specifically in the genus *Pseudobombax*, belonging to several chemical classes, such as alkaloids, flavonoids, phenolic compounds, and triterpenes [9,39,40].

Moreover, it is noteworthy that studies have reported that the anti-inflammatory and antioxidant effects of *Pseudobombax marginatum* and *P. ellipticum* species are closely related to their phenolic compound content. Overall, the ethnopharmacological interest in the genus *Pseudobombax* is focused on alcoholic preparations for its effectiveness in extracting and solubilizing their phytochemical constituents [41,42,43]. Furthermore, these studies represent pharmacological approaches that provide data for the safe application of the traditional use of medicinal plants in public health systems, as well as information to value traditional knowledge as a perspective for scientific/technological development [13,37,44].

In this regard, the *P. parvifolium*’s phytochemical composition displayed a phenolic compound content with intermediate values compared to data reported from other Malvaceae family species [45]. Studies with hydroalcoholic bark extracts of *P. marginatum* and *P. ellipticum* have reported concentrations greater than 80 mg EAG/g [10,40,46] compared to *P. parvifolium* (~30 mg EAG/g). These differences can be explained on the basis of edaphic factors, as well as seasonal and geographic variations, which affect the plants, determining alterations in the composition and concentration of secondary metabolites [47].

Hence, phytochemical analysis is relevant since several secondary metabolites are associated with biological properties resulting from their interactions with cellular biomolecules, arousing scientific interest in further studies on their therapeutic properties (e.g., antioxidant, anti-inflammatory, and antimicrobial effects) [16]. The EBHE qualitative analysis by LC–MS/MS showed the presence of catechin 7-arabinofuranoside, glycoside, and loliolide compounds, putatively identified. None of these compounds have yet been reported in *P. parvifolium*, for which phytochemical data are scarce. Coumarins, alkaloids, terpenes, and flavonoids have been identified as the main chemical compounds in other plant species from the subfamily Bombacoideae [41]. Loliolide, a monoterpenoid lactone, arouses therapeutic interest due to its various pharmacological effects, such as antiparkinsonian, antioxidant, and antitumoral [48,49]. Future experiments should be designed to validate the results.

In the cell viability screening, no cytotoxic effects were detected after exposure of 3T3, HEK293, VERO, and BRL-3A cells to EBHE. Cytotoxicity is a mandatory step applied as an in vitro approach to evaluate possible toxic effects of extracts and isolated molecules with potential human application [50].

Furthermore, health surveillance agencies require investigation of safety of plant extracts and their derivatives regarding possible mutagenic effects and DNA damage, aiming at their clinical application. The SMART assay using *D. melanogaster* is widely applied to investigate the mutagenicity of natural and synthetic compounds [51]. No direct or indirect mutagenic effects were observed in *D. melanogaster* somatic cells after exposure to different concentrations of EBHE, displaying even lower values for the total number of mutant clones compared to the negative control. The absence of mutagenicity is useful information considering the possibility of including EBHE in the primary human health care since the *Drosophila* genome possesses high homology with genes responsible for human diseases [26]. Hence, the corroboration of the absence of cytotoxicity and mutagenicity of natural products has become essential to guarantee the absence of risks regarding their potential clinical application [3,13].

Acute and repeated oral toxicity studies with EBHE also revealed no apparent toxicity signs or treatment-related mortality. Furthermore, feed and water intake and body weight were unaffected by EBHE administration over the observation period in both acute and recurrent oral toxicity. Results suggest no adverse effects after the administration of EBHE on the animal growth, metabolism, or clinical status. The EBHE displayed an LD_50_ greater than 2000 mg/kg in acute exposure, which according to the values of the Globally Harmonized System for Classification and Labeling of Chemicals (LD_50_ > 2000 mg/kg) is considered a safe [52]. Analysis of essential oils and plant extracts also demonstrated LD_50_ values >2000 mg/kg, indicating no toxic effects toward humans or the environment [53]. Therefore, these assays are mandatory to validate the use of plants in traditional folk medicine, which are widely marketed as safe products. The constant use of medicinal plants can cause harmful effects on human health, which stimulates evaluations to determine the potential toxicity of natural products [13,54].

Moreover, the weight of the excised organ after acute and repeated oral toxicity showed no significant change in treated animals compared to the control group, indicating once again the nontoxicity of EBHE. The assessment of organ weight is an index used repeatedly in toxicological studies in animal models since the organ weight decrease is indicative of toxicity after exposure to any substance [55]. The main organs affected by the induced metabolic reactions are the spleen, heart, liver, lungs, and kidneys. Among them, the liver is a critical target organ to assess toxicity due to the regulation of nutrient metabolism, as well as drug metabolism and detoxification [56].

Regarding liver function, no EBHE-induced increase in ALT and AST concentration was observed in the serum. These enzymes are indicators of liver toxicity, and their increase is due to changes in the permeability of hepatocytes or necrosis or cell damage [57]. Therefore, results suggest no toxic effect caused by EBHE since the ALT and AST values correspond to the normal physiological range established for Wistar rats [58]. Furthermore, results for ɣ-GT, ALP, and total protein excluded hepatotoxic effects due to EBHE exposure since their values were compatible with the control group.

Overall, exposure to xenobiotics can trigger reactions that promote cellular damage due to potentially toxic components, which are evaluated by the liver and kidneys since these organs are involved in drug metabolism and product excretion of normal metabolic processes, respectively [59]. Renal impairment is indicated by increased creatinine and urea concentrations due to low clearance of these substances [59]. In this regard, no change in serum uric acid concentration was observed, although urea concentration was significantly reduced in EBHE-treated animals compared to the control group. Nevertheless, this serum urea decrease is a positive indicator of renal function protection by the extract, probably due to the increase in diuresis, which is consistent with medicinal plants reportedly used to treat urolithiasis and inflammation, which also exhibited a significant decrease in urea content [30,60]. Again, experimental results showed no toxic effects of EBHE on kidney functions, supporting its therapeutic use in traditional medicine.

The significant reduction in TBARS concentration observed in EBHE-treated rats can be explained by a decrease in ROS production or an increase in the antioxidant enzyme activity, suggesting the inhibition of lipid peroxidation. The TBARS content decrease indicated no lipid peroxidation, probably due to the antioxidant effect of the polyphenols determined in EBHE’s chemical composition. This antioxidant capacity may be associated with flavonoid synergism, whose antioxidant potential can be increased through a polyphenol combination, acting at different levels on multiple targets and pathways, which provides benefits to improve treatment efficacy [61].

Furthermore, in the context of the EBHE antioxidant effect, no significant difference was observed comparing the GSH content between extract-treated and control animals. This intracellular antioxidant contributes to oxidative phosphorylation in the presence of an H_2_O_2_-generating system [54], and the antioxidant activity of plant extracts stimulates the cellular antioxidant defense system [62].

On the other hand, the significant increase in SOD activity in male rats treated with 400 mg/kg of EBHE suggests that the antioxidant function of this enzyme correctly acts against oxidative stress. These results indicate that EBHE and other plant extracts used in traditional medicine contain antioxidant compounds that may contribute to the health–disease balance [63]. SOD also plays an important role in decreasing the deleterious effect of free radicals on cells and their biomolecules [64].

GPx activity was evaluated only in repeated dose toxicity, since the administration of repeated dose toxicity is a more sensitive endpoint to evaluate a substance, providing extensive toxicological information, including clinical and histopathological signs [65]. The significant increased GPx activity at all administered EBHE doses indicated a protective effect by stimulating the enzymatic activity of the antioxidant immune system, with the concomitant hydroperoxide radicals and lipid superoxide reduction [66].

Although no organ was submitted to histopathological analysis, ALT and AST serum levels indicate no functional or structural alteration of liver cells since these alterations affect the physiological functioning of the organism. ALT and AST are widely trusted markers used clinically to determine liver injury and inflammation [66]. EBHE exposure under acute and repeated oral toxicity treatments caused positive effects, as indicated by biochemical (urea, uric acid, ALT, and AST) and antioxidant parameters (SD, GPx, GSH, and TBARS) analyses compared to control groups. Overall, these results indicated an absence of EBHE toxicity, as some of these parameters are part of the endogenous mechanism of antioxidant defense against oxidative stress by reducing superoxide radicals and lipid hydroperoxides, while alterations with other parameters are a consequence of the imbalance promoted by xenobiotic agents [62].

Regarding the EBHE’s antioxidant capacity, its phytochemical analysis revealed a reasonable polyphenol content, in addition to identifying the monoterpenoid lactone loliolide, which may be responsible for oxidative stress attenuation. Plant extracts and loliolide have been associated with anti-inflammatory responses, reducing/inhibiting oxidative damage, which protects cells against oxidation caused by ROS [48,67].

In vivo evaluation of triglycerides, cholesterol, and glucose after EBHE treatments of acute and repeated dose oral toxicity showed no significant difference between treated animals related to the control groups. Nonetheless, EBHE-treated rats displayed a slight reduction in serum triglyceride and glucose levels, evidencing extract hypoglycemic and hypolipidemic effects [30,67].

Additionally, mild hypoglycemic and hypolipidemic effects, as well as the decrease in urea levels evidenced after EBHE administration of acute and repeated oral doses, may be closely associated with polyphenols. In this framework, studies have highlighted the performance of these compounds as hypoglycemic agents by regulating blood glucose through the inhibition of α-glucosidase and the increase in the expression of the glucose transporter GLUT2 [68], while the hypolipidemic action may be related to restriction of lipid absorption due to bile acid chelation and inhibition of pancreatic lipase [69]. Overall, polyphenols have been reported as compounds with diverse pharmacological activities and research targets to develop drugs since they exert anti-inflammatory effects through radical-scavenging activities, regulation of cellular activities in inflammatory cells, and modulation of enzyme activities involved in metabolism arachidonic acid (phospholipase A2 and COX) and NO [62,70,71]. Furthermore, polyphenols can directly or indirectly inhibit or activate cellular and molecular processes via modulation of the expression of inflammatory mediators [72].

Toxicological tests with medicinal plants guarantee the safe use of traditional therapeutic resources due to the chemical diversity of plants. This requirement is mandatory considering the potential pharmaceutical applications of plants as their phytocomposition can trigger different responses in human health that are often unknown [2,73]. Moreover, this information is pivotal for the public health program development in several countries to provide plants recognized as medicinal, guaranteeing their quality and safety in serving the population and expanding the herbal medicine use [44,74].

It is noteworthy that the secondary metabolites evidenced in the genus *Pseudobombax* suggest a protective effect in experimental models; however, no plant extract should be used indiscriminately for long periods, since their diversity of chemical compounds can act synergistically or antagonistically, causing different pharmacological effects [61,75]. Therefore, the investigation of the pharmacological properties of *P. parvifolium* as a potential therapeutic resource should be explored to validate its use in traditional medicine, in addition to providing subsidies to insure its application in public health systems [74].

## 5. Conclusions

The hydroethanolic bark extract of *P. parvifolium* indicated a high polyphenol content, and the monoterpene lactone loliolide was putatively discovered for the first time in this species by LC–MS/MS analysis. No toxic effects of the extract were found in in vitro cytotoxicity tests, mutagenicity tests, and acute and repeated dosage oral toxicity experiments in Wistar rats. Additionally, there were no adverse effects of oxidative stress, cholesterol, triglycerides, glycemia, or liver and kidney function biochemical markers. Mild hypoglycemic and hypolipidemic effects were evidenced with repeated oral doses. Regarding the antioxidant parameters, data indicated a potential protective effect since the glutathione content showed no significant change, and SOD and GPx exhibited a significant increase at the dose of 400 mg/kg. These findings confirm *P. parvifolium*’s safe usage in conventional medicine and point to a possible alternative therapeutic use in the public health system. *P. parvifolium* may also be a viable source of bioactive chemicals. It could be necessary to do more studies to produce herbal medications using the *P. parvifolium* extract.

## Figures and Tables

**Table 1:** Phytocomponents identified in the *P. parvifolium* hydroalcoholic bark extract (EBHE) by LC–MS/MS analyses.

	Compound	Cosine	Mass Diffence	Mass	Molecular Formula	Ion Fragments	Adduct
**1**	Catechin 7-arabinofuranoside	0.87	0	423.13	C_20_H_22_O_10_	291.08, 165.05, 147.04, 139.04, 123.05	[M + H]^+^
**2**	Glycoside	0.88	0	600.265	C_28_H_38_O_13_	411.17, 249.11, 187.07, 131.08, 69.03	[M + NH_4_]^+^
**3**	Loliolide	0.87	0	197.116	C_11_H_16_O_3_	179.11, 161.09, 135.12, 133.10, 107.09	[M + H]^+^

**Table 2:** Results obtained with the *Drosophila melanogaster* in wing spot test (SMART) in the marker-heterozygous (MH) progeny of the standard (ST) and high bioactivation (HB) after chronic treatment of larvae with *P. parvifolium* hydroalcoholic bark extract (EBHE) and doxorubicin (DXR).

Genotypes and Treatments (mg/mL)	Number of Flies	Spots per Fly (Number of Spots) Estatistical Diagnosis ^a^												Spots with *Mwh* Clone ^c^ (*n*)
		Small Single Spots			Large Single Spots			Twin Spots			Total Spots			
		(1–2 Cells) ^b^			(>2 Cells) ^b^									
		*m* = 2			*m* = 5			*m* = 5			*m* = 2			
ST *														
**Negative Control**	40	0.28	(11)		0.05	(02)		0.00	(00)		0.33	(13)		13
**1.25**	40	0.20	(08)	−	0.00	(00)	i	0.00	(00)	i	0.20	(08)	−	8
**2.5**	40	0.25	(10)	i	0.00	(00)	i	0.00	(00)	i	0.25	(10)	−	10
**5**	40	0.33	(13)	i	0.00	(00)	i	0.03	(01)	i	0.35	(14)	i	14
**DXR (0.125)**	40	2.85	(114)	+	2.33	(93)	+	2.43	(97)	+	7.60	(304)	+	304
HB *														
**Negative Control**	40	0.45	(18)		0.05	(02)		0.00	(00)		0.50	(20)		20
**1.25**	40	0.35	(14)	−	0.03	(01)	i	0.00	(00)	i	0.38	(15)	−	15
**2.5**	40	0.38	(15)	−	0.05	(02)	i	0.05	(02)	i	0.48	(19)	−	19
**5**	40	0.45	(18)	−	0.08	(03)	i	0.03	(01)	i	0.55	(22)	−	22
**DXR (0.125)**	40	5.43	(217)	+	4.15	(166)	+	3.85	(154)	+	13.43	(537)	+	537

^*^ Marker-heterozygous flies (*mwh/flr^3^*) were evaluated. ^a^ Statistical diagnosis according to Frei and Würgler. U-test, two sided; probability levels: −, negative; +, positive; i, inconclusive; m, multiplication factor for significantly negative results. Level of significance *p* ≤ 0.05. ^b^ Including rare single spots *flr^3^*. ^c^ Considering *mwh* clones from *mwh* single and twin spots.

**Table 3:** Effects of *P. parvifolium* hydroalcoholic bark extract (EBHE) on relative organ weight in experiments of acute and repeated dose oral toxicity in Wistar rats.

Organ (g)	Acute Oral Toxicity		Repeated Dose Oral Toxicity							
	Control	2000 mg/kg	Control	100 mg/kg	200 mg/kg	400 mg/kg	Control	100 mg/kg	200 mg/kg	400 mg/kg
	Male		Female				Male			
**Kidney (R)**	0.40 ± 0.04	0.40 ± 0.05	1.45 ± 0.04	1.44 ± 0.16	1.42 ± 0.51	1.45 ± 0.01	1.38 ± 0.17	1.35 ± 0.13	1.30 ± 0.12	1.30 ± 0.05
**Kidney (L)**	0.41 ± 0.06	0.38 ± 0.05	1.36 ± 0.09	1.41 ± 0.11	1.45 ± 0.31	1.45 ± 0.01	1.25 ± 0.08	1.24 ± 0.13	1.30 ± 0.12	1.30 ± 0.10
**Liver**	3.41 ± 0.54	3.12± 0.32	9.54 ± 0.98	9.56 ± 0.56	9.45 ± 1.12	8.99 ± 1.32	8.54 ± 1.45	9.00 ± 1.43	8.00 ± 1.34	8.32 ± 1.42
**Spleen**	0.24 ± 0.04	0.18± 0.01	1.34 ± 0.54	1.35 ± 0.13	1.33 ± 0.44	1.47 ± 0.05	1.26 ± 0.25	1.28 ± 0.05	1.20 ± 0.01	1.27 ± 0.05
**Heart**	0.31 ± 0.02	0.30± 0.03	1.21 ± 0.02	1.24 ± 0.12	1.25 ± 0.05	1.30 ± 0.05	1.16 ± 0.17	1.14 ± 0.05	1.10 ± 0.02	1.03 ± 0.01
**Lung**	0.52 ± 0.07	0.55 ± 0.01	2.01 ± 0.54	1.99 ± 0.01	2.14 ± 0.01	1.49 ± 0.01	1.93 ± 0.30	1.78 ± 0.03	2.00 ± 0.14	1.80 ± 0.01
**Stomach**	0.95 ± 0.14	0.98 ± 0.05	3.12 ± 1.02	2.99 ± 0.02	2.90 ± 0.05	3.22 ± 0.45	3.18 ± 0.80	3.42 ± 0.78	3.35 ± 0.43	3.40 ± 0.33

Results are expressed as the mean ± SD. Acute oral toxicity (n = 3); repeated dose toxicity (n = 5). The control group was treated with vehicle (distilled water). Comparisons between groups were analyzed by means of ANOVA and Tukey’s post hoc test, providing no significant statistical results. R = right; L = left.

**Table 4:** Biochemical parameters of Wistar rats after treatment of acute and repeated dose oral toxicity with *P. parvifolium* hydroalcoholic bark extract (EBHE).

Biochemical Parameters	Acute Oral Toxicity		Repeated Dose Oral Toxicity							
	Control	2000 mg/kg	Control	100 mg/kg	200 mg/kg	400 mg/kg	Control	100 mg/kg	200 mg/kg	400 mg/kg
	Male		Female				Male			
**Gluc (mg/dL)**	122 ± 21.9	120 ± 1.30	124.34 ± 42.32	120.05 ± 22.12	122.09 ± 25.02	120.98 ± 17.32	121.73 ± 32.0	121.10 ± 16.13	115.43 ± 15.34	115.94 ± 12.31
**Trig (mg/dL)**	53 ± 5.65	55.6 ± 3.21	23.80 ± 6.87	24.99 ± 1.03	24.23 ± 1.09	23.98 ± 6.23	38.21 ± 5.12	36.12 ± 3.34	34.03 ± 1.76	35.22 ± 1.63
**Chol (mg/dL)**	54.5 ± 6.06	56.2 ± 0.18	64.63 ± 10.32	63.12 ± 2.43	64.09 ± 5.22	60.74 ± 3.98	60.21 ± 9.42	62.32 ± 7.02	60.04 ± 6.95	58.93 ± 4.58
**ALT (U/L)**	97 ± 4.24	99 ± 2.20	86.34 ± 15.64	88.32 ± 15.03	86.92 ± 13.33	85.65 ± 10.08	95.23 ± 11.54	96.65 ± 10.01	95.52 ± 8.98	93.13 ± 5.01
**AST (U/L)**	250 ± 23.3	225 ± 9.31	109.98 ± 14.39	110.65 ± 9.38	110.24 ± 6.39	110.76 ± 5.98	100.23 ± 23.3	100.32 ± 5.35	102.98 ± 1.54	100.76 ± 3.99
**γ-GT (U/L)**	12 ± 1.4	12.1 ± 0.02	10.98 ± 0.12	10.99 ± 1.05	10.12 ± 0.55	10.77 ± 1.01	9.89 ± 0.32	10.23 ± 1.61	10.32 ± 0.6	9.32 ± 0.12
**TB (mg/dL)**	0.38 ± 0.01	0.43 ± 0.01	1.46 ± 0.02	1.50 ± 0.01	1.40 ± 0.25	1.45 ± 0.24	1.35 ± 0.15	1.39 ± 0.01	1.35 ± 0.02	1.31 ± 0.43
**DB (mg/dL)**	0.10 ± 0.07	0.103 ± 0.05	1.37 ± 0.01	1.38 ± 0.05	1.38 ± 0.01	1.30 ± 0.05	1.43 ± 0.05	1.44 ± 0.01	1.40 ± 0.05	1.38 ± 0.12
**IB (mg/dL)**	0.28 ± 0.01	0.30 ± 0.09	0.09 ± 0.001	0.10 ± 0.001	0.08 ± 0.001	0.10 ± 0.005	0.08 ± 0.002	0.09 ± 0.001	0.08 ± 0.001	0.08 ± 0.001
**Urea (mg/dL)**	54.5 ± 7.7	30.2 ± 2.06 *	35.23 ± 7.21	25.87 ± 5.81 *	25.77 ± 4.81 *	20.54 ± 1.02 *	35.23 ± 7.21	24.87 ± 5.61 *	24.77 ± 2.81 *	20.22 ± 1.12 *
**Uric acid (mg/dL)**	1.16 ± 0.35	1.12 ± 0.01	1.15 ± 0.21	1.25 ± 0.08	1.26 ± 0.13	2.12 ± 0.01	1.16 ± 0.41	1.10 ± 0.01	1.13 ± 0.05	1.14 ± 0.05

Results are expressed as the mean ± SD. The control group was treated with vehicle (distilled water). Comparisons between groups were analyzed by ANOVA and Tukey’s post hoc test. Acute oral toxicity (n = 3); repeated dose toxicity (n = 5). Glucose (Gluc), triglycerides (Trig), cholesterol (Chol), alanine aminotransferase enzymes (ALT), aspartate aminotransferase (AST), gamma-glutamyl transferase (γ-GT), total bilirubin (TB), direct bilirubin (DB), indirect bilirubin (IB). * *p* < 0.05; values are significant compared to the control group of the same experiment.

## Data Availability

Not applicable.

## Supplementary Materials

The following supporting information can be downloaded at https://www.mdpi.com/article/10.3390/metabo13060748/s1: Comparison between library GNPS and query spectra of phytocomponents identified in *P. parvifolium* hydroalcoholic bark extract by LC–MS/MS analyses.

## References

[1] Zheng H., Fu X., Shao J., Tang Y., Yu M., Li L., Huang L., Tang K. (2023). Transcriptional regulatory network of high-value active ingredients in medicinal plants. Trends Plant Sci..

[2] Jităreanu A., Trifan A., Vieriu M., Caba I.-C., Mârţu I., Agoroaei L. (2023). Current trends in toxicity assessment of herbal medicines: A narrative review. Processes.

[3] Palhares R.M., Baratto L.C., Scopel M., Mügge F.L.B., Brandão M.G.L. (2021). Medicinal plants and herbal products from Brazil: How can we improve quality?. Front. Pharmacol..

[4] Santos M.O., Ribeiro D.A., Macêdo D.G., Macêdo M.J.F., Macedo J.G.F., Lacerda M.N.S., Macêdo M.S., Souza M.M.A. (2018). The Medicinal Plants: Versatility and concordance of use in the caatinga area, Northeastern Brazil. An. Acad. Bras. Cienc..

[5] Duarte M.C., Esteves G.L., Salatino M.L.F., Walsh K.C., Baum D.A. (2011). Phylogenetic analyses of Eriotheca and related genera (Bombacoideae, Malvaceae). Syst. Bot..

[6] Carvalho-Sobrinho J.G., Queiroz L.P. (2010). Three new species of Pseudobombax (Malvaceae, Bombacoideae) from Brazil. Novon.

[7] Albuquerque U.P., Medeiros P.M., Almeida A.L., Monteiro J.M., Neto E.M.F.L., Melo J.G., Santos J.P. (2007). Medicinal plants of the Caatinga (semi-arid) vegetation of NE Brazil: A quantitative approach. J. Ethnopharmacol..

[8] Das U., Islam M.S. (2019). A review study on different plants in Malvaceae family and their medicinal uses. Am. J. Biomed. Sci. Res..

[9] Paiva D.C., Santos C.A., Diniz J.C., Viana F.A., Thomazzi S.M., Falcão D.A. (2013). Anti-inflammatory and antinociceptive effects of hydroalcoholic extract from *Pseudobombax marginatum* inner bark from caatinga potiguar. J. Ethnopharmacol..

[10] Santos E.C.G., Donnici C.L., Camargos E.R.S., Araujo-Silva G., Xavier-Junior F.H., Farias L.M., Corrêa L.A., Luz J.R.D., Carvalho M.A.R., Almeida M.G. (2018). Interaction of *Pseudobombax marginatum* Robyns stem bark extract on the cell surface of *Bacillus cereus* and *Staphylococcus aureus*. J. Bacteriol. Mycol..

[11] Alves M.B.N., Barros N.B., Lugtenburg C.A.B., Barros R.R. (2022). Empirical use of medicinal plants in the treatment of diseases. Braz. J. Dev..

[12] Patrício K.P., Minato A.C.S., Brolio A.F., Lopes M.A., Barros G.R., Moraes V., Barbosa G.C. (2022). Medicinal plant use in primary health care: An integrative review. Ciên. Saúde Colet..

[13] Gaston T.E., Mendrick D.L., Paine M.F., Roe A.L., Yeung C.K. (2020). Natural is not synonymous with safe: Toxicity of natural products alone and in combination with pharmaceutical agents. Regul. Toxicol. Pharmacol..

[14] Kessler A., Kalske A. (2018). Plant secondary metabolite diversity and species interactions. Annu. Rev. Ecol. Evol. Syst..

[15] Twaij B.M., Hasan M.N. (2022). Bioactive secondary metabolites from plant sources: Types, synthesis, and their therapeutic uses. Int. J. Plant Biol..

[16] Aware C.B., Patil D.N., Suryawanshi S.S., Mali P.R., Rane M.R., Gurav R.G., Jadhav J.P. (2022). Natural bioactive products as promising therapeutics: A review of natural product-based drug development. S. Afr. J. Bot..

[17] Siddiqui A.J., Jahan S., Singh R., Saxena J., Ashraf S.A., Khan A., Choudhary R.K., Balakrishnan S., Badraoui R., Bardakci F. (2022). Plants in anticancer drug discovery: From molecular mechanism to chemoprevention. Biomed. Res. Int..

[18] ANVISA—National Health Surveillance Agency (2019). Brazilian Pharmacopoeia.

[19] Georgé S., Brat P., Alter P., Amiot M.J. (2005). Rapid determination of polyphenols and vitamin C in plant-derived products. J. Agric. Food Chem..

[20] Silva L.M.P., Inácio M.R.C., Silva G.G.C.d., Silva J.M.d.S.E., Luz J.R.D.d., Almeida M.d.G., Moraes E.P., Esposito D., Ferreira L.D.S., Zucolotto S.M. (2022). The first optimization process from cultivation to flavonoid-rich extract from *Moringa oleifera* Lam. leaves in Brazil. Foods.

[21] Luz J.R.D., Barbosa E.A., Nascimento T.E.S., Rezende A.A., Ururahy M.A.G., Brito A.S., Araujo-Silva G., López J.A., Almeida M.G. (2022). Chemical characterization of flowers and leaf extracts obtained from *Turnera subulata* and their immunomodulatory effect on LPS-activated RAW 264.7 macrophages. Molecules.

[22] Adan A., Kiraz Y., Baran Y. (2016). Cell proliferation and cytotoxicity assays. Curr. Pharm. Biotechnol..

[23] Kamiloglu S., Sari G., Ozdal T., Capanoglu E. (2020). Guidelines for cell viability assays. Food Front..

[24] Palhares L.C.G.F., Barbosa J.S., Scortecci K.C., Rocha H.A.O., Brito A.S., Chavante S.F. (2020). In vitro antitumor and anti-angiogenic activities of a shrimp chondroitin sulfate. Int. J. Biol. Macromol..

[25] Graf U., Würgler F.E., Katz A.J., Frei H., Juon H., Hall C.B., Kale P.G. (1984). Somatic mutation and recombination test in *Drosophila melanogaster*. Environ. Mol. Mutagen..

[26] Senes-Lopes T.F., López J.A., Amaral V.S., Brandão-Neto J., Rezende A.A., Luz J.R.D., Guterres Z.R., Almeida M.D.G. (2018). Genotoxicity of *Turnera subulata* and *Spondias mombin × Spondias tuberosa* extracts from Brazilian Caatinga biome. J. Med. Food.

[27] Efthimiou I., Vlastos D., Ioannidou C., Tsilimigka F., Drosopoulou E., Mavragani-Tsipidou P., Potsi G., Gournis D., Antonopoulou M. (2022). Assessment of the genotoxic potential of three novel composite nanomaterials using human lymphocytes and the fruit fly *Drosophila melanogaster* as model systems. Adv. Chem. Eng..

[28] ANVISA—National Health Surveillance Agency (2004). RE n° 90/2004. Standards for toxicological studies of herbal products. Brasília, DF: Official Gazette of the Federative Republic of Brazil, Executive Branch.

[29] OECD—Organisation for Economic Co-Operation and Development (2001). Test No. 423: Acute Oral Toxicity—Acute Toxic Class Method. OECD Guidelines for the Testing of Chemicals.

[30] Batista D., Luz J.R.D., Nascimento T.E.S., Senes-Lopes T.F., Galdino O.A., Silva S.V., Ferreira M.P., Azevedo M.A.S., Brandão-Neto J., Araujo-Silva G. (2021). *Licania rigida* leaf extract: Protective effect on oxidative stress, associated with cytotoxic, mutagenic and preclinical aspects. J. Toxicol. Environ. Health A.

[31] OECD—Organisation for Economic Co-Operation and Development (2008). Test No. 407: Repeated Dose 28-day Oral Toxicity Study in Rodents. OECD Guidelines for the Testing of Chemicals.

[32] Yagi K.A. (1976). A simple fluorometric assay for lipoperoxide in blood plasma. Biochem. Med..

[33] Beutler E., Duron O., Kelly B.M. (1963). Improved method for the determination of blood glutathione. J. Lab. Clin. Med..

[34] Frei H., Würgler F.E. (1988). Statistical methods to decide whether mutagenicity test data from Drosophila assay indicate a positive, negative, or inconclusive result. Mutat. Res..

[35] Kastenbaum M.A., Bowman K.O. (1970). Tables for determining the statistical significance of mutation frequencies. Mutat. Res..

[36] Kim J.K., Kim K.H., Shin Y.C., Jang B.-H., Ko S.-G. (2020). Utilization of traditional medicine in primary health care in low- and middle-income countries: A systematic review. Health Policy Plan..

[37] Theodoridis S., Drakou E.G., Hickler T., Thines M., Nogues-Bravo D. (2023). Evaluating natural medicinal resources and their exposure to global change. Lancet Planet. Health.

[38] Heinrich M. (2014). Ethnopharmacology: Quo vadis? Challenges for the future. Rev. Bras. Farmacogn..

[39] Menezes M.A.G., Neto F.B.O., Bertino L.M., Silva F.F.M., Alves L.A. (2015). Quantification of anthocyanins of embiratanha (*Pseudobombax marginatum*). Holos.

[40] Chaves T.P., Santana C.P., Véras G., Brandão D.O., Felismino D.C., Medeiros A.C.D., Trovão D.M.B.M. (2013). Seasonal variation in the production of secondary metabolites and antimicrobial activity of two plant species used in Brazilian traditional medicine. Afr. J. Biotechnol..

[41] Das G., Shin H.-S., Ningthoujam S.S., Talukdar A.D., Upadhyaya H., Tundis R., Das S.K., Patra J.K. (2021). Systematics, phytochemistry, biological activities and health promoting effects of the plants from the subfamily Bombacoideae (family Malvaceae). Plants.

[42] Refaat J., Desoky S.Y., Ramadan M.A., Kamel M.S. (2013). Bombacaceae: A phytochemical review. Pharm. Biol..

[43] Abubakar A.R., Haque M. (2020). Preparation of medicinal plants: Basic extraction and fractionation procedures for experimental purposes. J. Pharm. Bioallied Sci..

[44] Melro J.C.L., Fonseca S.A., Silva Júnior J.M., Franco S.P.B., Souza M.A., Costa J.G., Pimentel Y.F.C., Bomfim M.R.P., Almeida E.M., Matos-Rocha T.J. (2020). Ethnodirigid study of medicinal plants used by the population assisted by the “Programa de Saúde da Família” (Family Health Program) in Marechal Deodoro—AL, Brazil. Braz. J. Biol..

[45] Oliveira A.M.F., Pinheiro L.S., Pereira C.K.S., Matias W.N., Gomes R.A., Chaves O.S., Souza M.F.V., Almeida R.N., Assis T.S. (2012). Total phenolic content and antioxidant activity of some Malvaceae family species. Antioxidants.

[46] Ruiz-Terán F., Mendrano-Martinez A., Navarro-Ocaña A. (2008). Antioxidant and free radical scavenging activities of plant extracts used in traditional medicine in Mexico. Afric. J. Biotechnol..

[47] Pant P., Pandey S., Dall’Acqua S. (2021). The influence of environmental conditions on secondary metabolites in medicinal plants: A literature review. Chem. Biodivers..

[48] Han E.-J., Fernando I.P.S., Kim H.-S., Lee D.-S., Kim A., Je J.-G., Seo M.-J., Jee Y.-H., Jeon Y.-J., Kim S.-Y. (2021). (-)-Loliolide isolated from *Sargassum horneri* suppressed oxidative stress and inflammation by activating Nrf2/HO-1 signaling in IFN-γ/TNF-α-stimulated HaCaT keratinocytes. Antioxidants.

[49] Silva J., Alves C., Martins A., Susano P., Simões M., Guedes M., Rehfeldt S., Pinteus S., Gaspar H., Rodrigues A. (2021). Loliolide, a new therapeutic option for neurological diseases? In vitro neuroprotective and anti-inflammatory activities of a monoterpenoid lactone isolated from *Codium tomentosum*. Int. J. Mol. Sci..

[50] Di Nunzio M., Valli V., Tomás-Cobos L., Tomás-Chisbert T., Murgui-Bosch L., Danesi F., Bordoni A. (2017). Is cytotoxicity a determinant of the different in vitro and in vivo effects of bioactives?. BMC Complement. Altern. Med..

[51] Pitchakarn P., Inthachat W., Karinchai J., Temviriyanukul P. (2021). Human hazard assessment using Drosophila wing spot test as an alternative in vivo model for genotoxicity testing—A review. Int. J. Mol. Sci..

[52] UNECE—United Nations Economic Commission for Europe (2019). The Purple Book. Globally Harmonized System of Classification and Labelling of Chemicals (GHS).

[53] Ferraz C.A., Pastorinho M.R., Palmeira-de-Oliveira A., Sousa A.C.A. (2022). Ecotoxicity of plant extracts and essential oils: A review. Environ. Pollut..

[54] Sponchiado G., Adam M.L., Silva C.D., Soley B.S., Mello-Sampayo C., Cabrini D.A., Correr C.J., Otuki M.F. (2016). Quantitative genotoxicity assays for analysis of medicinal plants: A systematic review. J. Ethnopharmacol..

[55] Lazic S.E., Semenova E., Williams D.P. (2020). Determining organ weight toxicity with Bayesian causal models: Improving on the analysis of relative organ weights. Sci. Rep..

[56] Fitria L., Gunawan I.C.P., Sanjaya W.B.T., Meidianing M.I.M. (2022). Single-dose acute oral toxicity study of chloroform extract of snake plant (*Sansevieria trifasciata* Prain.) leaf in Wistar rats (*Rattus norvegicus* Berkenhout, 1769). J. Trop. Biodivers. Biotechnol..

[57] Gowda S., Desai P.B., Hull V.V., Math A.A.K., Vernekar S.N., Kulkarni S.S. (2009). A review on laboratory liver function tests. Pan Afr. Med. J..

[58] Guex C.G., Cassanego G.B., Dornelles R.C., Casoti R., Engelmann A.M., Somacal S., Maciel R.M., Duarte T., Borges W.S., Andrade C.M. (2022). Tucumã (*Astrocaryum aculeatum*) extract: Phytochemical characterization, acute and subacute oral toxicity studies in Wistar rats. Drug Chem. Toxicol..

[59] Croom E., Hodgson E. (2012). Metabolism of xenobiotics of human environments. Progress in Molecular Biology and Translational Science.

[60] Sansores-España D., Pech-Aguilar A.G., Cua-Pech K.G., Medina-Vera I., Guevara-Cruz M., Gutiérrez-Solis A.L., Reyes-García J.G., Avila-Nava A. (2022). Plants used in Mexican traditional medicine for the management of urolithiasis: A review of preclinical evidence, bioactive compounds, and molecular mechanisms. Molecules.

[61] Yuan H., Ma Q., Cui H., Liu G., Zhao X., Li W., Piao G. (2017). How can synergism of traditional medicines benefit from network pharmacology?. Molecules.

[62] Pisoschi A.M., Pop A., Iordache F., Stanca L., Predoi G., Serban A.E. (2021). Oxidative stress mitigation by antioxidants—An overview on their chemistry and influences on health status. Eur. J. Med. Chem..

[63] Rodríguez-Yoldi M.J. (2021). Anti-inflammatory and antioxidant properties of plant extracts. Antioxidants.

[64] Banerjee J., Das A., Sinha M., Saha S. (2018). Biological efficacy of medicinal plant extracts in preventing oxidative damage. Oxid. Med. Cell. Longev..

[65] Bulgheroni A., Kinsner-Ovaskainen A., Hoffmann S., Hartung T., Prieto P. (2009). Estimation of acute oral toxicity using the no observed adverse effect level (NOAEL) from the 28 day repeated dose toxicity studies in rats. Regul. Toxicol. Pharmacol..

[66] Ungur R.A., Borda I.M., Codea R.A., Ciortea V.M., Năsui B.A., Muste S., Sarpataky O., Filip M., Irsay L., Crăciun E.C. (2022). A Flavonoid-rich extract of *Sambucus nigra* L. reduced lipid peroxidation in a rat experimental model of gentamicin nephrotoxicity. Materials.

[67] Fu S., Wu D., Jiang W., Li J., Long J., Jia C., Zhou T. (2020). Molecular biomarkers in drug-induced liver injury: Challenges and future perspectives. Front. Pharmacol..

[68] Farias D.P., Araújo F.F., Neri-Numa A.A., Pastore G.M. (2021). Antidiabetic potential of dietary polyphenols: A mechanistic review. Food Res. Int..

[69] Feldman F., Koudoufio M., Desjardins Y., Spahis S., Delvin E., Levy E. (2021). Efficacy of polyphenols in the management of dyslipidemia: A focus on clinical studies. Nutrients.

[70] Hussain T., Tan B., Yin Y., Blachier F., Tossou M.C.B., Rahu N. (2016). Oxidative stress and inflammation: What polyphenols can do for us?. Oxid. Med. Cell. Longev..

[71] Al-Khayri J.M., Sahana G.R., Nagella P., Joseph B.V., Alessa F.M., Al-Mssallem M.Q. (2022). Flavonoids as potential anti-inflammatory molecules: A review. Molecules.

[72] Puangpraphant S., Cuevas-Rodríguez E.O., Oseguera-Toledo M., Levy N. (2022). Anti-inflammatory and antioxidant phenolic compounds. Current Advances for Development of Functional Foods Modulating Inflammation and Oxidative Stress.

[73] Dereli F.T.G., Thakur M., Belwal T. (2023). Plant-based bioactive components: Phytochemicals: A review. Bioactive Components.

[74] Silva J.D.N., Monção N.B.N., Farias R.R.S., Citó A.M.D.G.L., Chaves M.H., Araújo M.R.S., Lima D.J.B., Pessoa C., Lima A., Araújo E.C.D.C. (2020). Toxicological, chemopreventive and cytotoxic potentialities of rare vegetal species and supporting findings for the Brazilian Unified Health System (SUS). J. Toxicol. Environ. Health Part A.

[75] Rajčević N., Bukvički D., Dodoš T., Marin P.D. (2022). Interactions between natural products—A review. Metabolites.

