# Supplementary material for: Pseudobombax parvifolium Hydroalcoholic Bark Extract: Chemical Characterisation and Cytotoxic, Mutagenic, and Preclinical Aspects Associated with a Protective Effect on Oxidative Stress
Source: Metabolites. 2023 Jun 13;13(6):748. doi: 10.3390/metabo13060748 (PMC10302230; doi:10.3390/metabo13060748)

Supplementary material

Comparison between library GNPS and query spectra of phytocomponents identified by LC-MS/MS analyses

Comparison between library GNPS (bottom) and query spectra phytocomponents identified in **EBHE** (top). The structure of the phytocomponent identified is represented.

Catechin 7-arabinofuranoside

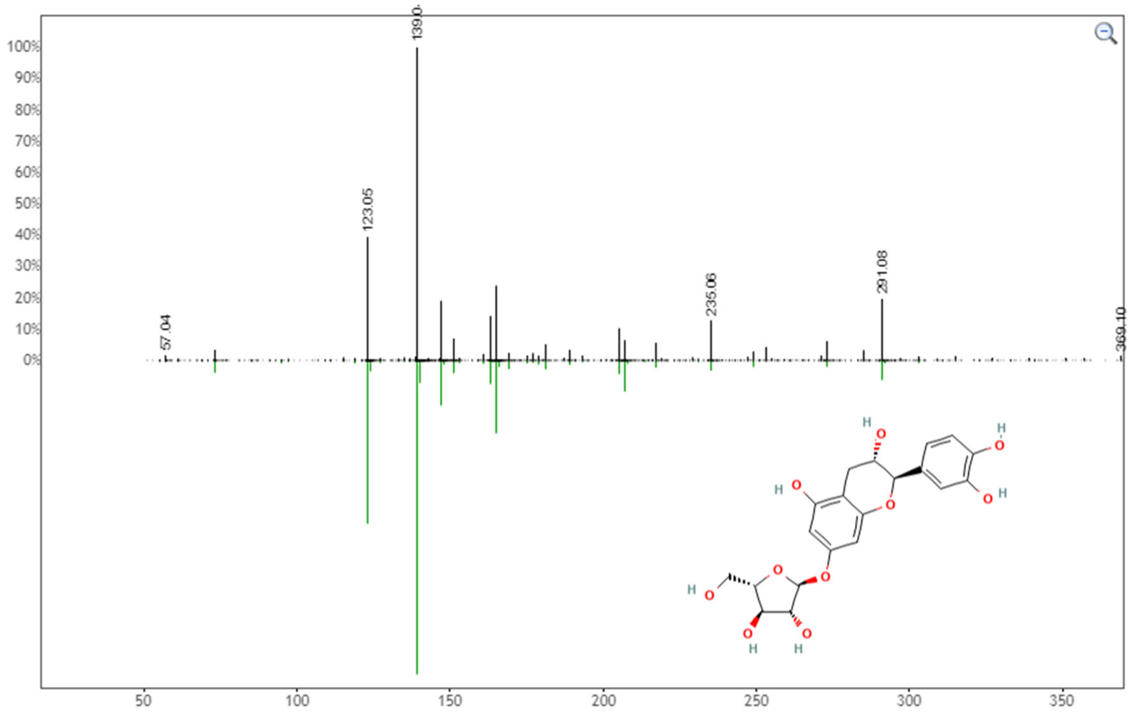

Glycoside: (NCGC00384749-012-[[5-(4-hydroxy-3,5-dimethoxyphenyl)-6,7-bis(hydroxymethyl)-1,3-dimethoxy-5,6,7,8-tetrahydronaphthalen-2-yl]oxy]-6-(hydroxymethyl)oxane-3,4,5-triol)

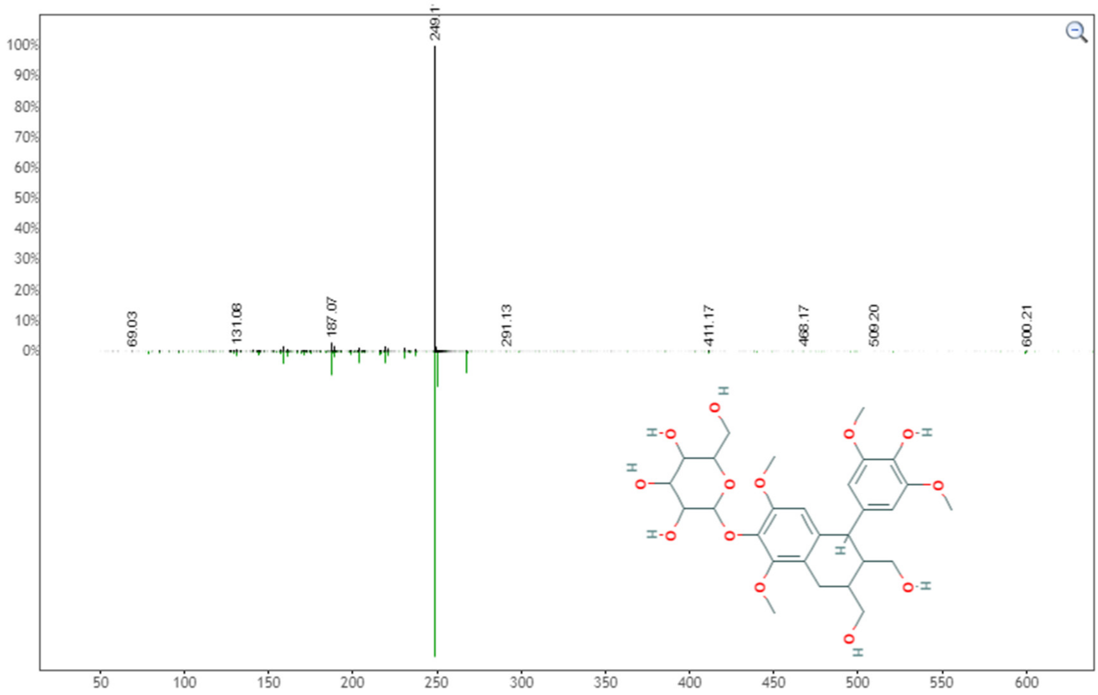

Loliolide

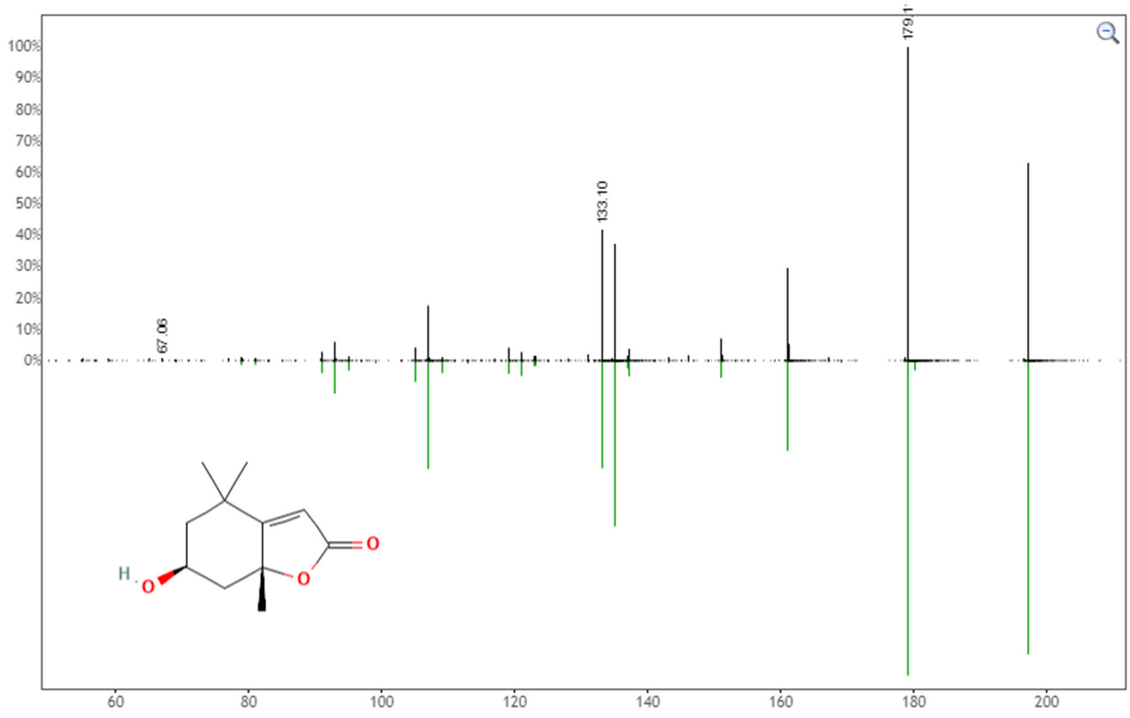

The whole chromatogram (until 10 min)

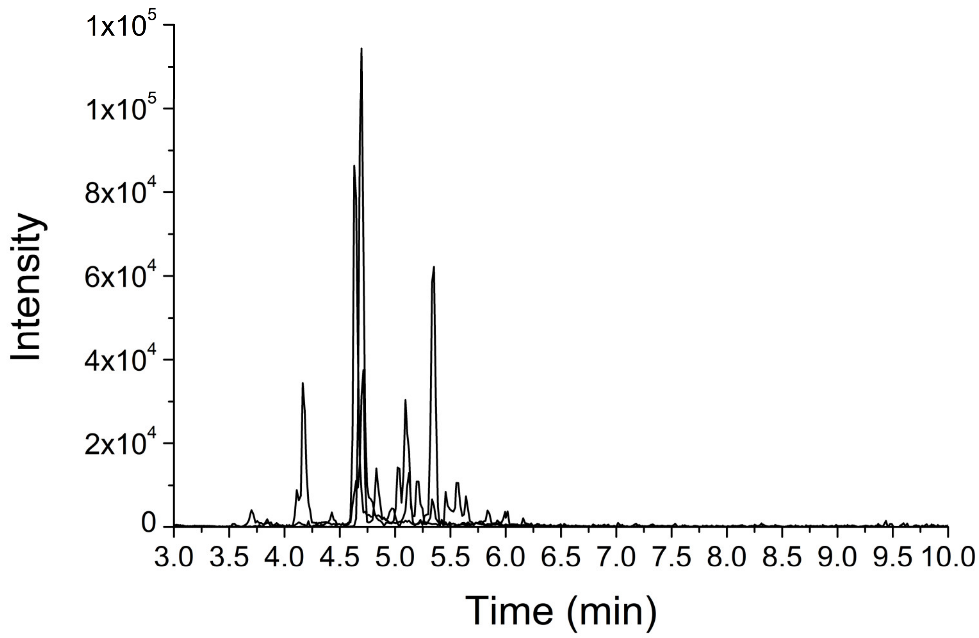

Supplement: Supplementary file 1 [file metabolites-13-00748-s001.zip › metabolites-2401487-supplementary.pdf]
